# Oxygen uptake kinetics and energy system’s contribution around maximal lactate steady state swimming intensity

**DOI:** 10.1371/journal.pone.0167263

**Published:** 2017-02-28

**Authors:** Jailton Gregório Pelarigo, Leandro Machado, Ricardo Jorge Fernandes, Camila Coelho Greco, João Paulo Vilas-Boas

**Affiliations:** 1 University Catholic Center of Quixadá–UNICATÓLICA, Quixadá, Ceará, Brazil; 2 Metropolitan College of Grande Fortaleza–FAMETRO, Fortaleza, Ceará, Brazil; 3 Centre of Research, Education, Innovation and Intervention in Sport, Faculty of Sport, University of Porto, Porto, Portugal; 4 Porto Biomechanics Laboratory, LABIOMEP, University of Porto, Porto, Portugal; 5 Human Performance Laboratory, Physical Education Department, São Paulo State University, Rio Claro, São Paulo, Brazil; Universitat de Valencia, SPAIN

## Abstract

The purpose of this study was to examine the oxygen uptake ($\dot{V}O_{2}$) kinetics and the energy systems’ contribution at 97.5, 100 and 102.5% of the maximal lactate steady state (MLSS) swimming intensity. Ten elite female swimmers performed three-to-five 30 min submaximal constant swimming bouts at imposed paces for the determination of the swimming velocity (v) at 100%MLSS based on a 7 x 200 m intermittent incremental protocol until voluntary exhaustion to find the v associated at the individual anaerobic threshold. $\dot{V}O_{2}$ kinetics (cardiodynamic, primary and slow component phases) and the aerobic and anaerobic energy contributions were assessed during the continuous exercises, which the former was studied for the beginning and second phase of exercise. Subjects showed similar time delay (TD) (mean = 11.5–14.3 s) and time constant (τ_p_) (mean = 13.8–16.3 s) as a function of v, but reduced amplitude of the primary component for 97.5% (35.7 ± 7.3 mL.kg.min^-1^) compared to 100 and 102.5%MLSS (41.0 ± 7.0 and 41.3 ± 5.4 mL.kg.min^-1^, respectively), and τ_p_ decreased (mean = 9.6–10.8 s) during the second phase of exercise. Despite the slow component did not occur for all swimmers at all swim intensities, when observed it tended to increase as a function of v. Moreover, the total energy contribution was almost exclusively aerobic (98–99%) at 97.5, 100 and 102.5%MLSS. We suggest that well-trained endurance swimmers with a fast TD and τ_p_ values may be able to adjust faster the physiological requirements to minimize the amplitude of the slow component appearance, parameter associated with the fatigue delay and increase in exhaustion time during performance, however, these fast adjustments were not able to control the progressive fatigue occurred slightly above MLSS, and most of swimmers reached exhaustion before 30min swam.

## Introduction

An important aspect of aerobic endurance performance is the ability to sustain the highest percentage of maximal oxygen uptake ($\%\dot{V}O_{2\max}$) as long as possible. In this sense, coaches and swimmers have used the $\%\dot{V}O_{2\max}$ in different submaximal intensities to control, prescribe and improve sports training [1]. Additionally, scientists have shown that the $\dot{V}O_{2}$ kinetics analysis may help to understand the physiological adjustments produced over time by the athletes in several sports, allowing them to maintain a high $\%\dot{V}O_{2\max}$ in a physiological steady-state during aerobic endurance performance [2–4].

Meanwhile, the scientific community has mainly described the $\dot{V}O_{2}$ kinetics in three different intensity domains during continuous exercise. First, the moderate domain is described as the exercise intensities in which a state steady for $\dot{V}O_{2}$ is achieved within 3 min of constant exercise [5]. Subsequently, the heavy domain is described as the exercise intensities in which $\dot{V}O_{2}$ slow component should be evident, causing a delay on the achievement of the $\dot{V}O_{2}$ steady-state during exercise [2]. Last, the severe domain is described as the exercise intensities in which $\dot{V}O_{2}$ is elevated compared to rest values and continue to increase over time, leading to attain the $\dot{V}O_{2\max}$ [6, 7].

Maximal lactate steady state (MLSS) is considered one of the main relevant parameters for prescription and improvement of aerobic endurance performance, once it has been assumed as the limit intensity at which, during prolonged and submaximal exercise, the metabolic energy is produced mainly by the aerobic metabolism of pyruvate and glycolysis [8, 9]. Moreover, MLSS is identified as the maximal intensity that can be maintained over time without the lactate production exceeding removal more than 1 mmol.L^-1^, and considered gold-standard method for the evaluation of aerobic capacity [10–12].

Once maximal velocity where a steady-state is found represents a fundamental physiological border, subtle changes in this intensity could likely modify $\dot{V}O_{2}$ kinetics response. For instance, when the exercise is performed at intensities slightly below MLSS, a physiological steady state is sustained for both blood lactate concentration [La-] and $\dot{V}O_{2}$ as a function of time [6, 7, 13]. On the other hand, at intensities above the MLSS, a significant increase in [La-] and $\dot{V}O_{2}$ is likely to be observed throughout time [3, 7, 8, 12], leading to fatigue and voluntary exhaustion [3, 4, 14]. Moreover, the swimming MLSS determination needs a short time of interruption for the blood collection during the 10^th^ minute of exercise for the analysis of [La-], and then, a resumption of exercise to complete the test. Thus, it seems to be fundamental to examine the behavior of $\dot{V}O_{2}$ kinetics not only the beginning of exercise, but too after the resumption of exercise throughout exercise to better understanding of the entire process of the swimmer physiological response along the exercise.

$\dot{V}O_{2}$ kinetics has been studied in different sports over the last decades [2, 6, 15], and there are relevant number of researches based on [La-] and gas exchange at intensities related to MLSS [8, 13, 14]. However, no study has evaluated $\dot{V}O_{2}$ kinetics at (and around) the MLSS intensity. Thus, our purpose was to examine $\dot{V}O_{2}$ kinetics and the energy systems’ contribution at 97.5, 100 and 102.5%MLSS in swimming. It was hypothesized that at 97.5%MLSS, $\dot{V}O_{2}$ kinetics adjustments may not be so evident such as 100 and 102.5%MLSS. It was further hypothesized that even at the 100%MLSS intensity, swimmers may also have to adjust $\dot{V}O_{2}$ kinetics during the exercise, once this intensity would lead to voluntary exhaustion over time. On the other hand, at the intensity of 102.5%MLSS, $\dot{V}O_{2}$ kinetics may be compromised by fatigue, requiring faster time adjustments for time delay and time constant, and higher $\dot{V}O_{2}$ amplitudes either for primary or slow components compared to lower exercise intensities. We further intended to assess $\dot{V}O_{2}$ kinetics of the second phase of exercise, starting after the collection of [La-] and resumption of exercise (from 10^th^ min to the exercise end—final exercise), hypothesizing that these parameters could be faster than without previous exercise. Moreover, as MLSS may be maintained for long time period without continuous [La-] accumulation, as well as a submaximal exercise, the energy supply should be mainly supported through the aerobic system for the swimming intensities of ± 2.5% around MLSS.

## Material and methods

Ten elite female swimmers volunteered and gave written informed consent (or parent/guardian when subjects were under 18yrs) to participate in the present study, which was approved by the Ethics Committee of Faculty of Sport from the University of Porto and performed according to the Declaration of Helsinki. The swimmers were (mean ± SD) 17.6 ± 1.9 years of age, 1.70 ± 0.05 m height, 61.3 ± 5.8 kg body mass, 15.5 ± 2.9% body fat mass, and 54.9 ± 6.7 mL.kg.min^-1^ $\dot{V}O_{2\max}$, specialized in middle- and long-distance swimming events. The subjects had, at the least, seven years of experience as competitive swimmers and their mean performance over a 400m freestyle swim was 88.0 ± 3.4% of the short course word record.

The test sessions were performed in a 25 m indoor swimming pool. Air humidity was maintained nominally between 40–60%, and pool water temperature between 27–28°C. Swimmers were advised to refrain from intense training at least 24 h before the experimental sessions. The tests were conducted within a seven day period, at the same time of the day (± 2 h), minimizing the circadian rhythm effects. Previously to the test sessions, swimmers performed a 1000 m warm-up at low/moderate intensity. The tests were performed in front crawl, with in-water starts and open turns, without relevant underwater glides. A 24 h interval was imposed between all tests.

Initially, swimmers performed an intermittent incremental protocol until voluntary exhaustion to find the velocity (v) corresponding to the individual anaerobic threshold (IAnT). The distance covered in each step was 200 m, with v increases of 0.05 m.s^-1^ and 30 s rest intervals between each swim [16]. According to these authors, the predetermined v of the last step was defined as the currently best expected performance for the subjects’ 400 m front crawl, and then used to define all the v steps for the incremental test. The IAnT was assessed by the relationship between [La-] and v using a curve fitting method, and considered the interception point between linear and exponential regressions to determine the accurate v where [La-] increased exponentially [16, 17].

Subsequently, each swimmer performed three-to-five 30 min submaximal constant swimming bouts at imposed paces to determine the highest v where a MLSS was achieved (100%MLSS). The first trial was performed at the v corresponding to IAnT; and, if a steady state or a decrease in [La-] was observed, further subsequent trials with 2.5% higher velocities were performed until no [La-] steady state could be maintained [14]. Following this study, if the first trial resulted in a clearly identifiable increase of the [La-], and/or could not be sustained due to exhaustion, further trials were conducted with reduced velocities. MLSS was defined as the [La-] that increased by no more than 1 mmol.l^-1^ between the 10^th^ and 30^th^ min of the test [9].

Earlobe capillary blood samples (5 μL) were collected: (a) at rest and in the first 30 s after each step of the incremental test, immediately after exhaustion, and at each 2 min of recovery (until the [La-] recovery peak was found); and (b) at rest, 10 and 30^th^ min (or voluntary exhaustion) of each continuous bout (Lactate Pro, Arkray, Inc., Kyoto, Japan).

The v was set and maintained using a visual underwater pacer (GBK-Pacer, GBK Electronics, Aveiro, Portugal), with lights located each 2.5 m apart by a light strip on the bottom of the pool. Swimmers followed the flashing lights to maintain the predetermined velocities. and were instructed to keep their heads above each visual signal. Exhaustion was defined when the swimmers remained 5 m behind the lights.

$\dot{V}O_{2}$ was measured by a telemetric portable gas analyzer (K4b^2^, Cosmed, Italy) in both tests, connected to the swimmer by a low hydrodynamic resistance respiratory snorkel and valve system (New AquaTrainer^®^, Cosmed, Italy). This system has been previously validated [18] and used in similar studies [15]. The device was calibrated for minute ventilation ($\dot{V}E$) with a calibrated syringe (3 L) and the O_2_ and CO_2_ analyzers with standard calibration gases (16% O_2_ and 5% CO_2_) before each test. In all tests, $\dot{V}O_{2}$ data were analyzed and errant breaths occurred by swallow water and/or saliva, sighs and coughs were excluded. Afterwards, $\dot{V}O_{2}$ values were measured in mean ± 3 SD and outside values were removed. Subsequently, the breath-by-breath data were linearly interpolated to provide five-by-five s values, and smoothed using three breath averages [15, 19]. Heart rate (HR) was monitored and registered continuously by a HR monitor system (Polar Vantage NV, Polar electro Oy, Kempele, Finland) and transferred in real time, through a telemetric signal, to the K4b^2^ device. The HR values were also averaged every 5 s intervals.

The average $\dot{V}O_{2}$ values were analyzed by a nonlinear least squares algorithm to fit the data through MatLab 7.0 Software (MathWorks, Natick, MA). The mathematical model consisted of two (cardiodynamic and primary components) or three (cardiodynamic, primary and slow components) exponential models. An F-Test (p < 0.05) was used to evaluate whether the two or three exponentials models provided the best fit to each data set.

$$\begin{array}{l} \dot{V}O_{2}\;(t)=\dot{V}O_{2\;\mathrm{baseline}}+A_{c}\;[1–e^{\text{-}(t/\tau }{{}_{c}}^{)}]\;\mathrm{Phase}\;I\;(\mathrm{cardiodynamic}\;\mathrm{component})\\ \;+A_{p}\;[1–e^{\text{-}(t\text{-}\mathrm{TD}}{{}_{p}}^{)}{{}^{/\tau }}_{p}]\;\mathrm{Phase}\;\mathrm{II}\;(\mathrm{primary}\;\mathrm{component})\\ \;+A_{s}\;[1–e^{\text{-}(t\text{-}\mathrm{TD}}{{}_{s}}^{)/\tau }{}_{s}]\;\mathrm{Phase}\;\mathrm{III}\;(\mathrm{slow}\;\mathrm{component}) \end{array}$$

where $\dot{V}O_{2}$ (t) represents the absolute $\dot{V}O_{2}$ at time, $\dot{V}O_{2\;\mathrm{baseline}}$ is the $\dot{V}O_{2}$ in resting baseline period, A_c_ and τ_c_ are the amplitude and the time constant of the cardiodynamic component; A_p_, TD_p_ and τ_p_ are the amplitude, the time delay and the time constant of the primary component; A_s_, TD_s_ and τ_s_ are the amplitude, the time delay and the time constant of the slow component. The mean response time (MRT) was applied to represent the overall pulmonary $\dot{V}O_{2}$ kinetics response, which was determined as the sum of TD_p_ and τ_p_ [15]. The $\dot{V}O_{2}$ kinetics was assessed during the beginning of exercise until the break (at the 10^th^ min) of swim for collection of [La-] (initial exercise), and the second phase of exercise, starting after the collection of [La-] and resumption of exercise (final exercise).

The energy systems’ contribution has been assessed by the total energy expenditure ($\dot{E}$). The $\dot{E}$ was obtained by the addition of the aerobic energy expenditure calculated by the difference between the exercise $\dot{V}O_{2}$ ($\dot{V}O_{2\mathrm{exercise}}$) and baseline $\dot{V}O_{2}$ ($\dot{V}O_{2\mathrm{baseline}}$) (mL.kg^-1^.min^-1^), and by the anaerobic energy expenditure that was calculated by the net [La-] values transformed into O_2_ equivalents using the constant value of 2.7 mLO_2_.kg^-1^.mM^-1^ [15, 20] during continuous exercises.

Data are presented as mean and standard deviation (± SD). Normality and sphericity of data were checked with the Shapiro-Wilk’s W and Mauchley Sphericity tests. When the assumption of sphericity was not attained, Greenhouse-Geisser or the Huynh-Feld adjusted univariate tests for repeated measures were used. The partial Eta square (ή_p_^2^) was used to measure the effect size, defined as small, medium and large for values of 0.01, 0.06 and 0.14, respectively [21]. The comparisons of $\dot{V}O_{2}$ kinetics (cardiodynamic and primary components) and energy systems’ contribution (aerobic and anaerobic energy expenditure) were performed using multivariate ANOVA and examined by the intensity and previous exercise effects. The v and [La-] values were performed using the univariate ANOVA. All analyses were conducted for repeated measures, complemented with the Bonferroni correction post-hoc test with a significance level of p < 0.05.

## Results

All swimmers performed 30 min when swimming at 97.5 and 100%MLSS, but eight swimmers were not able to maintain the predetermined v during 30 min at 102.5%MLSS, reaching voluntary exhaustion at 19.3 ± 4.9 min. The average v and $\%\dot{V}O_{2\max}$ values were different in between the three swim intensities, with 97.5%MLSS slowest and lowest, and 102.5%MLSS fastest and highest (*F*_2,18_ = 2560.200, *p* < 0.001, ή_p_^2^ = 0.996; *F*_2,18_ = 15.538, *p* < 0.001, ή_p_^2^ = 0.633, respectively) (Table 1). [La-] and HR values for the three swim intensities are also shown in Table 1 with a higher values at 102.5%MLSS compared to 97.5 and 100%MLSS for [La-] (*F*_2,18_ = 18.123, *p* < 0.001, ή_p_^2^ = 0.668), and at 102.5%MLSS compared to 97.5%MLSS for HR (*F*_2,18_ = 7.222, *p* < 0.005, ή_p_^2^ = 0.445).

**Table 1:** Mean (SD) values of swimming velocity (v), blood lactate concentrations ([La-]), heart rate (HR), and percentage of maximal oxygen uptake ($\%\dot{V}O_{2\max}$) are shown at 97.5, 100 and 102.5% of the maximal lactate steady state (MLSS) (N = 10).

	97.5%MLSS	100%MLSS	102.5%MLSS
v (m.s^-1^)	1.21 (0.07)	1.24 (0.07)^1^	1.27 (0.07)^1^^,^^2^
[La-] (mmol.L^-1^)	1.48 (0.39)	1.89 (0.77)	2.97 (0.87)^1^^,^^2^
HR (beats.min^-1^)	167.1 (15.0)	173.6 (9.7)	179.3 (9.2)^1^
$\%\dot{V}O_{2\max}$ (%)	78.9 (8.7)	84.7 (3.8)^1^	90.9 (4.6)^1^^,^^2^

^1,2^ Values different from 97.5 and 100%MLSS, respectively (p < 0.05).

$\dot{V}O_{2}$ kinetics parameters are presented in Table 2. A_p_ tended to increase with the swimming intensity (v) during the initial exercise, but differences were only noticed comparing 100 and 102.5%MLSS to 97.5%MLSS (*F*_2,18_ = 8.249, *p* < 0.05, ή_p_^2^ = 0.478). Meanwhile, A_p_ was similar at final exercise for the three swim conditions (*F*_2,18_ = 1.167, *p* = 0.334, ή_p_^2^ = 0.115). On the other hand, A_p_ decreased as a function of previous exercise for the three swims bouts. TD_p_, τ_p_ and MRT were similar as function of v at initial exercise and final exercise during the three swimming conditions. However, when analyzed the swim bouts as a function of previous exercise, TD_p_ decreased for the 97.5%MLSS, but the values remained similar for 100 and 102.5%MLSS; τ_p_ decreased for all swim intensities, and MRT decreased for the 97.5 and 102.5%MLSS, but remained similar for 100%MLSS.

**Table 2:** Mean (SD) values of $\dot{V}O_{2}$ kinetics parameters at velocities of 97.5, 100 and 102.5% of the maximal lactate steady state (MLSS) for the beginning of exercise until the break of swim for blood collection (initial exercise), and the second phase of exercise, starting after blood collection (final exercise) (N = 10).

	97.5%MLSS		100%MLSS		102.5%MLSS	
	Initial exercise	Final exercise	Initial exercise	Final exercise	Initial exercise	Final exercise
VO_2 baseline_ (mL.kg^-1^.min^-1^)	7.2 (2.1)	16.0 (5.3)^a^	6.0 (1.0)	17.4 (5.7)^a^	6.4 (0.8)	18.8 (5.8)^a^
A_c_ (mL.kg^-1^.min^-1^)	16.4 (5.9)	10.4 (4.9)^a^	16.1 (7.1)	14.2 (5.4)	15.1 (6.5)	14.9 (5.7)
A_p_ (mL.kg^-1^.min^-1^)	35.7 (7.3)	26.3 (7.4)^a^	41.0 (7.0)^1^	28.3 (5.2)^a^	41.3 (5.4)^1^	29.8 (5.5)^a^
TD_p_ (s)	14.3 (5.5)	12.0 (5.3)^a^	12.4 (8.1)	11.9 (4.9)	11.5 (6.8)	11.1 (4.7)
τ_p_ (s)	16.3 (5.4)	10.8 (4.7)^a^	13.8 (4.5)	9.7 (4.5)^a^	16.0 (5.8)	9.6 (5.3)^a^
MRT (s)	30.6 (5.2)	22.8 (5.4)^a^	26.2 (6.8)	21.6 (4.6)	27.4 (8.5)	20.7 (5.2)^a^

Statistical analyses were described by intensity and previous exercise effect.

^1^ Values different from 97.5%MLSS for initial exercise.

^a^ Values different from initial exercise (*p* < 0.05).

The both measured $\dot{V}O_{2\mathrm{baseline}}$ at initial exercise (*F*_2,18_ = 2.389, *p* = 0.120, ή_p_^2^ = 0.210) and final exercise (*F*_2,18_ = 1.034, *p* = 0.376, ή_p_^2^ = 0.103) were similar in between the three swim conditions, but $\dot{V}O_{2\mathrm{baseline}}$ increased as a function of previous exercise (initial to final exercise) for all continuous intensities (*F*_1,9_ = 68.311, *p* < 0.001, ή_p_^2^ = 0.884). A_c_ was similar as a function of v for both initial exercise (*F*_2,18_ = 0.134, *p* = 0.876, ή_p_^2^ = 0.015) and final exercise (*F*_2,18_ = 1.974, *p* = 0.168, ή_p_^2^ = 0.180). Moreover, at 97.5%MLSS, A_c_ was lower comparing initial and final exercise, but values remained similar for 100 and 102.5%MLSS.

A_s_ of $\dot{V}O_{2}$ kinetics was observed for all tested swimming intensities and testing phases (initial and final exercise) only in two out of ten subjects. In one subject A_s_ was not observed. The A_s_ was observed for 6 swimmers during initial exercise and 8 swimmers during final exercise at 97.5%MLSS, for 6 swimmers during initial exercise and 7 swimmers during final exercise at 100%MLSS, and for 9 swimmers during initial exercise and 5 swimmers during final exercise at 102.5%MLSS. The A_s_ values are presented in Table 3. A_s_ tended to increase with swimming intensity during initial exercise, but keeping constant during final exercise whatever the intensity considered; however no statistical analysis was applied, once the occurrence of the A_s_ was apparently chaotic among swimmers both considering swimming intensities and phases of testing (initial and final exercise).

**Table 3:** Individual and mean (SD) values of the amplitude of slow component (A_s_) at velocities of 97.5, 100 and 102.5% of the maximal lactate steady state (MLSS) for the beginning of exercise until the break of swim for blood collection (initial exercise), and the second phase of exercise, starting after blood collection (final exercise) (N = 10).

	A_s_ (mL.kg^-1^.min^-1^)					
	97.5%MLSS		100%MLSS		102.5%MLSS	
swimmer	Initial exercise	Final exercise	Initial exercise	Final exercise	Initial exercise	Final exercise
1	1.7	2.9	2.3	3.8	4.5	1.6
2	2.3	0.7	4.4	0	3.7	0
3	1.1	0	2.6	0	4.4	0
4	0	0	0	0	0	0
5	4.2	0.9	2.8	0.8	2.9	0
6	0	0.8	0	0.8	1.9	2.2
7	0	1.2	0	0.9	7.2	1.1
8	0	1.7	2.8	1.1	4.5	0
9	2.5	1.3	2.6	1.5	6.1	0.8
10	1.4	0	0	1.5	5.1	1.8
Mean (SD)	2.2 (1.1)	1.4 (0.8)	2.9 (0.8)	1.5 (1.1)	4.5 (1.6)	1.5 (0.6)

**Fig 1:**
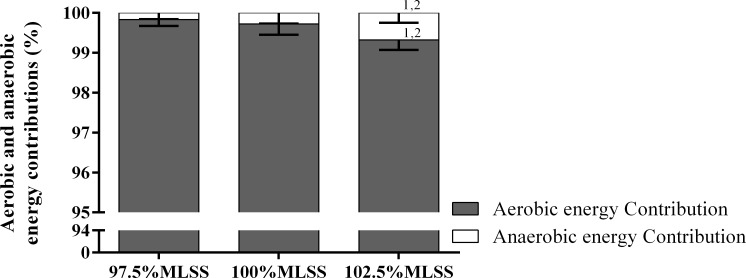
Mean ± SD of aerobic and anaerobic energy relative contribution values at velocities corresponding to 97.5, 100 and 102.5% of the maximal lactate steady state (MLSS).

The relative energy contribution for each one of the three swim intensity bouts is shown in Fig 1. The aerobic energy contribution decreased (*F*_2,18_ = 15.254, *p* < 0.001, ή_p_^2^ = 0.629) and the anaerobic energy increased (*F*_2,18_ = 15.254, *p* < 0.001, ή_p_^2^ = 0.629) at 102.5%MLSS compared to 97.5 and 100%MLSS.

## Discussion

The purposes of this study were to examine the $\dot{V}O_{2}$ kinetics responses during constant-velocity swims at intensities of 97.5, 100 and 102.5%MLSS, the effect of previous exercise on the parameters of $\dot{V}O_{2}$ kinetics, and the contribution of the energetic systems at the three conditions. The main original findings were that increasing exercise intensity resulted in greater primary amplitude of the $\dot{V}O_{2}$ kinetics, in accordance with previous results in running [22]. As demonstrated by other studies [23–25], the previous exercise may increase the amplitude of the primary component and accelerate $\dot{V}O_{2}$ kinetics (i.e., MRT) during the subsequent exercise. There was a significant increase in the anaerobic contribution when swimming above MLSS. However, the aerobic energetic system contribution corresponded to ~99% of the total energy demand of the exercise in all exercise conditions analyzed in this study.

In sports science, $\dot{V}O_{2}$ kinetics have added the understanding of physiological adjustments over time [2–4], such as muscle metabolism and systemic oxygen transport [26]. Moreover, one of the most relevant exercise intensities in swimming for aerobic training, prescription and evaluation is the v at which MLSS is obtained, being considered the direct and gold-standard method for the evaluation of aerobic capacity [8, 10–12, 14]. Thus, both aspects ($\dot{V}O_{2}$ kinetics and MLSS) are decisive for the understanding of energy supply and oxidative metabolism supporting muscular exercise. Therefore, our purpose was to examine the amplitude and time adjustments of $\dot{V}O_{2}$ kinetics during swims at intensities of 97.5, 100 and 102.5%MLSS, exploring the effects of small prescriptions variations on swimming oxidative physiology.

The main findings were: (a) A_p_ tended to increase with swimming v for the initial phase of exercise, despite differences were only noticed comparing 100 and 102.5%MLSS to 97.5%MLSS. Meanwhile, A_p_ was similar at the final phase of exercise during the three swim conditions. However, A_p_ decreased as a function of previous exercise for the three swim intensities; (b) TD_p_, τ_p_ and MRT were similar irrespective of v both at initial and final exercise; (c) regarding the effect of previous exercise comparing initial and final exercise for the three swimming intensities, TD_p_ decreased for the 97.5%MLSS, but was similar for 100 and 102.5%MLSS, τ_p_ decreased for all swim intensities, and MRT decreased for the 97.5 and 102.5%MLSS, but was similar for 100%MLSS; (d) although A_s_ was not evident for all swimmers during the three swimming conditions, it tended to increase with intensity during initial exercise, remaining constant during final exercise; (e) A_c_ was similar both for the initial and final exercise comparing the three swim intensities, but was lower during final exercise compared to initial exercise at 97.5%MLSS, and was similar at 100 and 102.5%MLSS; (f) aerobic and anaerobic energy contributions were different at 102.5%MLSS compared to lower swim velocities; (g) at the three swim intensities, the aerobic contribution values were higher than 98% of the total energy input.

The $\dot{V}O_{2}$ values in the present study were directly measured breath-by-breath throughout time for the three swim intensities. Subsequently, the $\dot{V}O_{2}$ data were fitted through mathematical modelling as previously applied in swimming for maximal and submaximal exercises [15, 19, 27–30]. Some studies have reported $\dot{V}O_{2}$ kinetics at intensities near the maximal v where a steady state in swimming is found (MLSS) [27–29], however we are unaware of a study that has evaluated and compared $\dot{V}O_{2}$ kinetics at or around the MLSS in swimming. Most of previous studies reported in sports science [2, 15, 19, 27–31] have studied $\dot{V}O_{2}$ kinetics at maximal and submaximal intensities, demonstrating the fundamental role of $\dot{V}O_{2}$ kinetics to understand the physiological mechanisms underpinning the dynamics of the aerobic response at different exercise intensities. Thus, the understanding of the $\dot{V}O_{2}$ kinetics throughout time may aid the evaluation of aerobic capacity and prescription of specific training sets during these fundamental training intensities around MLSS.

The 100%MLSS v values reported in this study are in accordance with those reported in previous ones [13, 14, 32], in spite of the fact that most of the swimmers examined in the previous studies were male when compared with the female subjects of the present study. Despite higher v values at a given relative intensity are expected to be higher for male than female counterparts of similar training level [33], the sex similitude comparing our results with literature could likely be explained by a higher technical and biomechanical proficiency of our female swimmers when compared to the male swimmers of the previous studies. Indeed, the $\%\dot{V}O_{2\max}$ at 100%MLSS (85 ± 4%) observed in the present study for women is similar to previously reported data for men (86.1% $\dot{V}O_{2\mathrm{peak}}$) [34], suggesting similar levels of aerobic capacity development, even the $\dot{V}O_{2\max}/\dot{V}O_{2\mathrm{peak}}$ being higher in the previous study (mean = ~83 mL.kg^-1^.min^-1^) when compared with our results (54.9 ± 6.7 mL.kg^-1^.min^-1^). Meanwhile, the mean HR value at 100%MLSS was 174 ± 10 beats.min^-1^ in the present study, values which were similar to the previous reported in literature [32, 34], as expected by the comparable age of samples.

Moreover, the [La-] at 100%MLSS (1.89 ± 0.77 mmol.L^-1^) in the present study were lower when compared to swimming literature (2.8–3.3 mmol.L^-1^) [14, 34, 35]. These lower [La-] values may be explained by sex differences for similar levels of aerobic capacity development, with expected lower values for women due to lower body mass and lean muscle mass compared to men [36]. Furthermore, women have showed lower testosterone concentration compared to men [37] during aerobic endurance exercise [33, 36], suggesting different metabolic contributions between carbohydrates and fat during long-distance exercise [33, 38], and supporting comparable lower [La-].

Since the early research on $\dot{V}O_{2}$ kinetics [39] until up to date, the time constant (τ) has been studied in sports science in the attempt to comprehend the physiological adjustments during the non-steady state period at the beginning of exercise due to the increase of metabolic demand. In the present study, the τ_p_ values were similar between intensity levels for the initial exercise phase (mean = 15.4 ± 5.2 s) and final exercise phase (mean = 10.0 ± 4.7 s), but the values decreased with previous exercise for the three swim conditions. This is particularly relevant for training practice, underlining the influence of previous exercise on the subsequent metabolic dynamics. In all studied exercise intensities, the τ_p_ in the present study showed similar values compared than those previously reported in swimming (~15–20 s) [27–29], cycling [40, 41], rowing [42], and running [43, 44]. Thus, those values reported for intensities up to and above the MLSS seem to behave similarly as expected, based on the previous knowledge on the $\dot{V}O_{2}$ kinetics during different intensity domains for well-trained athletes. Indeed, a faster attainment of a steady state and a reduction in the oxygen deficit are associated to the fatigue delay and increase in exhaustion time, being well trained athletes able to perform at higher intensities with lower requirements of anaerobic energy during the transition from rest to exercise [5]. Hence, the lower τ_p_ values reported in this study when compared to previously published ones regarding physiological adaptations induced by aerobic endurance training confirm the highly endurance training status and specialization (endurance athletes) of our swimmers [5, 44].

Partially in contrast with previous literature that showed the existence of the A_s_ at these exercise intensities [2, 4, 5], in the present study it has shown to occur chaotically during the three swimming conditions, with very diverse individual occurrence profiles; however, observing the sample data a tendency to A_s_ increase as a function of intensity was observed (2.2 ± 1.1, 2.9 ± 0.8 and 4.5 ± 1.6 mL.kg^-1^.min^-1^, respectively for 97.5, 100 and 102.5%MLSS), but only during initial exercise, not during the final phase after metabolic adaptation already occurred. Besides, only two swimmers showed A_s_ occurrence in all trials both at the initial and final exercise phases, and one swimmer did not show any A_s_ during all the swimming efforts and phases. It is worthy to emphasize the curiosity of that particular swimmer being a national record holder (800 and 1500m) and the best endurance swimmer of the sample. These partially contradictory findings could be explained, at least in part, by the specific physiological adaptations occurred through the highly endurance training status for our swimmers, such as faster τ_p_ [44], possible increase in the mitochondrial content of the cell [45], beyond also possible alterations in the mitochondrial sensitivity to the respiration regulators [46], and the fact of these endurance athletes might have mainly type I muscle fibers [45]. Thus, our endurance swimmers with fast $\dot{V}O_{2}$ kinetics would be able to adjust faster the physiological requirements for aerobic performance during the high intensity aerobic exercises, minimizing the A_s_ demand. In addition, the appearance of the A_s_ is normally explained by a phenomena that may be attenuated in our very specialized sample, namely the recruitment of type II fibers with fatigue [47], after which the magnitude of A_s_ has been correlated with the rise of [La-] [2, 45]. Thereby, the absence of significant A_s_ in the present study may be likely explained by the high-level of endurance training of the sample [48].

Moreover, to reinforce the predominance of aerobic energy system during the three swim conditions around MLSS, the present study determined the total energy contribution at each one of the studied exercise intensities. At all swimming intensities up to and above MLSS, the aerobic energy contribution was higher than 98% of the total energy contribution; however there were significant differences between the highest and the lower v regarding aerobic and anaerobic energy contributions. This study was the first study to show the energy contribution during intensities at and around MLSS directly measured breath-by-breath in swimming, which highlights that even at intensities above MLSS; the total energy contribution was mainly and almost exclusively controlled by the oxidative bioenergetics system.

## Conclusions

The present study showed that well-trained endurance swimmers with a fast component of $\dot{V}O_{2}$ kinetics, i.e. an abrupt and fast increase in $\dot{V}O_{2}$ response, and low [La-] may be able to adjust faster the physiological requirements during intensities up to and slightly above MLSS to minimize the appearance of the slow component of $\dot{V}O_{2}$ and reduce the oxygen deficit, both parameters are associated to the fatigue delay and the increase in exhaustion time, key factors to endurance performance. however, these fast adjustments were not able to control the progressive fatigue occurred slightly above MLSS, and most of swimmers reached exhaustion before 30min swam. Moreover, the data shows that the aerobic energy contribution at intensities up to and slightly above MLSS plays a fundamental role controlling almost exclusively the required energy supply.

## Supporting information

S1 File Values of physiological parameters at 97.5, 100 and 102.5% of the maximal lactate steady state (MLSS) (N = 10). (PDF)

S2 FileValues of $\dot{V}O_{2}$ kinetics parameters at 97.5, 100 and 102.5% of the maximal lactate steady state (MLSS) (N = 10).(PDF)
