# Supplementary material for: Oxygen uptake kinetics and energy system’s contribution around maximal lactate steady state swimming intensity
Source: PLoS One. 2017 Feb 28;12(2):e0167263. doi: 10.1371/journal.pone.0167263 (PMC5330462; doi:10.1371/journal.pone.0167263)
Supplement: S1 File — (PDF) [file pone.0167263.s001.pdf]

97,5% MLSS	0%	0%	0%	0%	0%	0%	4min	4min	4min	4min	4min	4min	25%	25%	25%	25%	25%	25%	33%
	VE	VO2	VCO2	VO2/Kg	R	HR	VE	VO2	VCO2	VO2/Kg	R	HR	VE	VO2	VCO2	VO2/Kg	R	HR	VE
	l/min	ml/min	ml/min	ml/min/Kg	---	bpm	l/min	ml/min	ml/min	ml/min/Kg	---	bpm	l/min	ml/min	ml/min	ml/min/Kg	---	bpm	l/min
Swimmer 1	13,5	414,9	394,9	6,3	1,0	66,8	51,0	2366,6	1946,4	35,9	0,8	143,9	53,1	2260,6	1982,3	34,3	0,9	147,2	53,6
Swimmer 2	11,7	368,5	343,3	5,8	0,9	64,5	67,9	2692,6	2466,1	42,1	0,9	172,4	68,4	2665,1	2464,4	41,6	0,9	180,9	65,1
Swimmer 3	14,3	397,4	395,0	7,4	1,0	86,5	68,7	3025,4	2568,5	56,0	0,8	167,9	70,3	2852,3	2554,5	52,8	0,9	172,7	73,8
Swimmer 4	24,1	394,4	582,3	7,0	1,5	91,3	69,9	2792,3	2385,0	49,9	0,9	185,6	69,6	2788,8	2422,6	49,8	0,9	196,0	68,0
Swimmer 5	9,6	312,7	299,6	5,6	1,0	71,6	63,8	2718,5	2428,0	48,5	0,9	166,0	68,1	2817,2	2577,6	50,3	0,9	166,0	66,4
Swimmer 6	9,8	328,3	263,7	5,9	0,8	63,6	61,8	2330,0	2095,9	41,6	0,9	162,9	64,8	2309,1	2114,8	41,2	0,9	159,3	62,9
Swimmer 7	22,9	815,1	691,0	12,7	0,9	92,1	56,6	2653,0	2091,1	41,5	0,8	153,3	58,0	2589,3	2140,4	40,5	0,8	152,3	59,0
Swimmer 8	14,2	517,2	455,2	7,2	0,9	82,3	47,4	2281,8	1980,4	31,7	0,9	127,6	43,3	2237,2	1915,4	31,1	0,9	149,8	45,4
Swimmer 9	15,1	502,9	426,8	7,7	0,9	90,0	66,8	3044,8	2577,4	46,8	0,8	169,8	75,7	3152,1	2759,7	48,5	0,9	175,3	72,9
Swimmer 10	11,9	376,3	262,8	6,3	0,7	69,7	69,8	2931,6	2152,7	48,9	0,7	154,8	64,8	2654,7	2055,4	44,2	0,8	152,4	66,1
MEAN	14,7	442,8	411,5	7,2	0,94	77,8	62,4	2683,7	2269,2	44,3	0,8	160,4	63,6	2632,7	2298,7	43,4	0,87	165,2	63,3
DP	5,0	146,4	137,9	2,1	0,21	11,7	8,1	280,8	241,5	7,2	0,1	16,3	9,6	294,3	291,3	7,1	0,0	15,9	8,7

100% MLSS	0%	0%	0%	0%	0%	0%	4min	4min	4min	4min	4min	4min	25%	25%	25%	25%	25%	25%	33%
	VE	VO2	VCO2	VO2/Kg	R	HR	VE	VO2	VCO2	VO2/Kg	R	HR	VE	VO2	VCO2	VO2/Kg	R	HR	VE
	l/min	ml/min	ml/min	ml/min/Kg	---	bpm	l/min	ml/min	ml/min	ml/min/Kg	---	bpm	l/min	ml/min	ml/min	ml/min/Kg	---	bpm	l/min
Swimmer 1	13,79	370,58	429,88	5,61	1,16	75,67	72,13	3042,59	2688,52	46,10	0,88	167,33	68,19	2841,20	2584,59	43,05	0,91	169,58	66,71
Swimmer 2	13,83	377,43	357,56	5,90	0,94	61,13	62,04	2915,39	2420,11	45,55	0,83	164,58	64,55	2970,72	2546,70	46,42	0,86	172,42	63,15
Swimmer 3	15,27	444,79	430,23	8,24	0,97	89,00	73,64	2933,25	2724,70	54,32	0,93	174,83	78,97	2951,16	2794,82	54,65	0,95	182,58	81,70
Swimmer 4	9,69	291,91	285,48	5,21	0,98	87,00	77,52	3174,44	2659,72	56,69	0,84	185,50	76,54	3020,31	2606,85	53,93	0,86	190,67	76,28
Swimmer 5	10,38	299,00	328,25	5,34	1,11	88,29	66,05	2871,12	2620,54	51,27	0,91	171,17	73,61	3058,88	2791,46	54,62	0,91	177,33	70,25
Swimmer 6	10,31	365,65	283,20	6,53	0,77	73,67	57,87	2346,35	1970,56	41,90	0,84	168,17	60,74	2335,05	2032,72	41,70	0,87	167,33	61,31
Swimmer 7	12,41	395,16	316,19	6,17	0,80	66,58	62,40	2775,27	2241,20	43,36	0,81	157,08	62,40	2775,27	2241,20	43,36	0,81	157,08	64,71
Swimmer 8	11,49	376,70	337,57	5,23	0,90	63,45	61,64	2895,43	2467,70	40,21	0,85	163,58	62,78	2883,27	2540,75	40,05	0,88	169,55	55,62
Swimmer 9	13,05	413,18	380,95	6,36	0,93	63,22	84,00	3100,94	2855,07	47,71	0,92	171,75	85,25	3128,77	2975,14	48,13	0,95	181,75	80,19
Swimmer 10	11,07	292,81	239,71	4,88	0,83	61,57	68,26	2801,06	2270,28	46,68	0,81	165,00	74,16	2868,83	2448,80	47,81	0,85	165,00	70,57
MEAN	12,13	362,72	338,90	5,95	0,94	72,96	68,56	2885,58	2491,84	47,38	0,86	168,90	70,72	2883,35	2556,30	47,37	0,89	173,33	69,05
DP	1,84	52,47	62,50	0,97	0,12	11,51	8,21	228,04	270,59	5,30	0,05	7,66	8,23	220,18	274,12	5,49	0,04	9,84	8,43

102.5% MLSS	0%	0%	0%	0%	0%	0%	4min	4min	4min	4min	4min	4min	25%	25%	25%	25%	25%	25%	33%
	VE	VO2	VCO2	VO2/Kg	R	HR	VE	VO2	VCO2	VO2/Kg	R	HR	VE	VO2	VCO2	VO2/Kg	R	HR	VE
	l/min	ml/min	ml/min	ml/min/Kg	---	bpm	l/min	ml/min	ml/min	ml/min/Kg	---	bpm	l/min	ml/min	ml/min	ml/min/Kg	---	bpm	l/min
Swimmer 1	13,09	449,30	376,76	6,81	0,84	69,75	76,43	3315,86	2873,08	50,24	0,87	173,75	83,92	3311,52	2986,70	50,17	0,90	180,67	80,47
Swimmer 2	15,24	406,82	407,61	6,36	1,01	65,60	65,13	2980,44	2590,57	46,57	0,87	172,50	66,83	3024,01	2706,99	47,25	0,90	177,83	67,25
Swimmer 3	11,29	362,87	281,86	6,72	0,77	74,00	89,56	3154,63	2796,33	58,42	0,89	173,58	85,79	3078,74	2725,10	57,01	0,89	170,08	90,05
Swimmer 4	25,91	390,33	599,12	6,97	1,54	86,50	81,64	3234,78	2775,31	57,76	0,86	192,18	79,62	3135,62	2755,10	55,99	0,88	199,00	82,27
Swimmer 5	10,35	290,60	320,72	5,19	1,09	94,33	72,33	2981,41	2766,05	53,24	0,93	170,92	75,07	3029,07	2825,73	54,09	0,93	181,92	75,96
Swimmer 6	13,05	377,45	360,18	6,74	0,95	57,36	70,04	2541,92	2295,68	45,39	0,90	168,75	72,87	2566,93	2343,23	45,84	0,91	174,00	68,29
Swimmer 7	14,72	417,38	381,27	6,52	0,91	80,83	78,58	2990,85	2552,96	46,73	0,85	171,00	79,29	3034,43	2599,56	47,41	0,86	171,83	79,37
Swimmer 8	10,85	370,64	304,82	5,15	0,82	61,42	73,38	3113,18	2614,83	43,24	0,84	175,00	71,76	3079,26	2571,28	42,77	0,84	175,00	68,62
Swimmer 9	15,85	504,46	434,19	7,76	0,86	81,80	73,23	3155,90	2788,84	48,55	0,88	176,08	74,65	3156,15	2828,80	48,56	0,90	177,00	77,78
Swimmer 10	15,60	361,68	327,73	5,98	0,91	69,38	76,25	3062,68	2417,72	50,62	0,79	156,08	73,19	3124,15	2464,11	51,64	0,79	161,18	74,44
MEAN	14,60	393,15	379,42	6,42	0,97	74,10	75,66	3053,16	2647,14	50,08	0,87	172,98	76,30	3053,99	2680,66	50,07	0,88	176,85	76,45
DP	4,46	57,25	90,46	0,80	0,22	11,65	6,69	211,40	186,55	5,09	0,04	8,79	5,81	191,12	189,78	4,60	0,04	9,77	7,17

33%	33%	33%	33%	33%	50%	50%	50%	50%	50%	50%	66%	66%	66%	66%	66%	66%	75%	75%	75%	75%
VO2	VCO2	VO2/Kg	R	HR	VE	VO2	VCO2	VO2/Kg	R	HR	VE	VO2	VCO2	VO2/Kg	R	HR	VE	VO2	VCO2	VO2/Kg
ml/min	ml/min	ml/min/Kg	---	bpm	l/min	ml/min	ml/min	ml/min/Kg	---	bpm	l/min	ml/min	ml/min	ml/min/Kg	---	bpm	l/min	ml/min	ml/min	ml/min/Kg
2242,3	1985,4	34,0	0,9	147,4	51,8	2189,9	1911,6	33,2	0,9	146,3	51,4	2166,1	1877,4	32,8	0,9	145,3	54,7	2295,8	1984,6	34,8
2510,0	2341,2	39,2	0,9	180,9	59,4	2528,6	2289,3	39,5	0,9	184,6	64,4	2563,7	2355,8	40,1	0,9	187,3	63,0	2536,6	2342,3	39,6
2909,6	2641,3	53,9	0,9	175,8	71,4	2852,2	2533,6	52,8	0,9	174,8	71,6	2892,9	2561,3	53,6	0,9	175,5	73,3	2922,2	2567,6	54,1
2742,7	2410,8	49,0	0,9	194,3	68,9	2750,0	2411,7	49,1	0,9	196,1	66,3	2664,6	2334,6	47,6	0,9	196,0	69,9	2749,6	2420,5	49,1
2805,7	2551,3	50,1	0,9	167,0	64,5	2708,3	2469,3	48,4	0,9	172,9	66,4	2631,2	2435,9	47,0	0,9	162,4	69,4	2705,0	2536,2	48,3
2265,5	2094,3	40,5	0,9	165,2	65,0	2265,1	2112,8	40,4	0,9	166,7	65,3	2281,2	2098,1	40,7	0,9	168,0	63,7	2265,8	2055,8	40,5
2571,1	2130,9	40,2	0,8	154,1	58,5	2500,9	2054,4	39,1	0,8	152,0	59,4	2545,0	2094,9	39,8	0,8	152,9	60,0	2598,2	2122,9	40,6
2295,8	1993,4	31,9	0,9	151,8	46,3	2233,3	1991,7	31,0	0,9	152,0	48,6	2256,0	2034,1	31,3	0,9	150,2	49,2	2442,2	2121,6	33,9
3032,5	2594,0	46,7	0,9	174,6	69,5	2925,8	2486,8	45,0	0,8	172,3	76,2	3114,5	2659,5	47,9	0,9	176,1	78,0	3143,1	2668,4	48,4
2714,3	2040,1	45,2	0,8	154,8	74,1	2840,1	2177,3	47,3	0,8	166,1	77,1	2823,4	2218,2	47,1	0,8	170,7	80,4	2808,5	2240,5	46,8
2609,0	2278,3	43,1	0,9	166,6	62,9	2579,4	2243,9	42,6	0,9	168,4	64,7	2593,9	2267,0	42,8	0,9	168,4	66,2	2646,7	2306,0	43,6
278,7	259,6	7,1	0,1	14,9	8,9	276,3	225,0	7,1	0,0	15,4	9,4	301,4	246,8	7,1	0,0	16,2	10,0	276,3	236,8	6,7

33%	33%	33%	33%	33%	50%	50%	50%	50%	50%	50%	66%	66%	66%	66%	66%	66%	75%	75%	75%	75%
VO2	VCO2	VO2/Kg	R	HR	VE	VO2	VCO2	VO2/Kg	R	HR	VE	VO2	VCO2	VO2/Kg	R	HR	VE	VO2	VCO2	VO2/Kg
ml/min	ml/min	ml/min/Kg	---	bpm	l/min	ml/min	ml/min	ml/min/Kg	---	bpm	l/min	ml/min	ml/min	ml/min/Kg	---	bpm	l/min	ml/min	ml/min	ml/min/Kg
2827,32	2531,27	42,84	0,90	171,08	70,12	2952,59	2634,14	44,74	0,89	173,83	72,13	3053,25	2729,82	46,26	0,89	176,33	71,96	3048,10	2712,34	46,18
2901,74	2497,48	45,34	0,86	173,58	61,99	2882,23	2439,64	45,03	0,85	173,08	64,26	2913,49	2506,80	45,52	0,86	173,50	62,34	2881,68	2458,01	45,03
2916,23	2759,68	54,00	0,95	184,42	80,49	2830,92	2656,11	52,42	0,94	184,50	80,77	2733,79	2567,16	50,63	0,94	183,58	80,27	2722,05	2554,29	50,41
2948,36	2630,34	52,65	0,89	194,33	71,52	2857,92	2509,36	51,03	0,88	191,75	73,06	3013,99	2767,21	53,82	0,92	194,33	70,29	2864,99	2645,72	51,16
3001,33	2678,50	53,60	0,89	179,17	66,70	2882,75	2558,43	51,48	0,89	176,92	69,73	2923,41	2651,43	52,20	0,91	181,75	67,52	2803,30	2547,33	50,06
2337,86	2056,19	41,75	0,88	168,67	64,50	2287,93	2030,48	40,86	0,89	167,00	67,65	2291,83	2052,55	40,93	0,90	164,50	58,16	2432,14	1961,31	43,43
2725,63	2331,59	42,59	0,86	160,92	64,43	2728,58	2283,50	42,63	0,84	160,33	66,33	2665,91	2264,53	41,65	0,85	161,25	65,34	2684,29	2230,57	41,94
2626,70	2226,30	36,48	0,85	158,82	55,94	2666,27	2336,44	37,03	0,88	164,83	59,03	2739,24	2380,83	38,04	0,87	169,33	60,46	2889,50	2428,55	40,13
3051,70	2886,70	46,95	0,95	181,67	75,43	2840,37	2635,80	43,70	0,93	172,92	81,84	2903,35	2788,16	44,67	0,96	177,83	82,47	2966,25	2797,18	45,63
2807,02	2385,71	46,78	0,85	165,00	72,35	2831,07	2387,76	47,18	0,84	165,00	79,77	2819,78	2460,82	47,00	0,87	165,00	75,89	2845,98	2376,35	47,43
2814,39	2498,38	46,30	0,89	173,77	68,35	2776,06	2447,17	45,61	0,88	173,02	71,46	2805,80	2516,93	46,07	0,90	174,74	69,47	2813,83	2471,17	46,14
210,19	253,88	5,75	0,04	11,19	7,10	189,52	196,27	4,97	0,03	9,59	7,58	219,36	236,47	5,08	0,04	10,20	8,26	170,97	244,14	3,70

33%	33%	33%	33%	33%	50%	50%	50%	50%	50%	50%	66%	66%	66%	66%	66%	66%	75%	75%	75%	75%
VO2	VCO2	VO2/Kg	R	HR	VE	VO2	VCO2	VO2/Kg	R	HR	VE	VO2	VCO2	VO2/Kg	R	HR	VE	VO2	VCO2	VO2/Kg
ml/min	ml/min	ml/min/Kg	---	bpm	l/min	ml/min	ml/min	ml/min/Kg	---	bpm	l/min	ml/min	ml/min	ml/min/Kg	---	bpm	l/min	ml/min	ml/min	ml/min/Kg
3341,42	3049,45	50,63	0,91	183,17	79,72	3241,26	2841,47	49,11	0,88	182,50	88,40	3390,00	3025,39	51,36	0,89	186,42	87,28	3336,51	2976,60	50,55
2966,54	2700,27	46,35	0,91	182,75	64,41	2880,63	2480,72	45,01	0,86	183,92	66,15	2862,99	2559,54	44,73	0,89	184,00	66,40	2914,14	2638,48	45,53
3150,33	2791,58	58,34	0,89	175,00	98,06	3153,99	2841,98	58,41	0,90	178,25	96,75	3024,82	2645,98	56,02	0,87	181,92	99,41	3139,50	2688,20	58,14
3247,06	2903,55	57,98	0,89	202,80	82,47	3023,98	2639,39	54,00	0,87	202,25	80,56	3082,09	2687,66	55,04	0,87	202,33	83,27	3076,27	2708,17	54,93
3062,80	2841,17	54,69	0,93	177,92	72,78	3043,06	2734,27	54,34	0,90	181,64	73,50	2894,09	2661,58	51,68	0,92	181,67	71,91	2854,38	2614,29	50,97
2520,16	2271,71	45,00	0,90	174,83	72,94	2489,02	2299,62	44,45	0,92	180,00	77,87	2545,57	2345,17	45,46	0,92	182,58	78,72	2566,66	2301,03	45,83
3008,38	2585,44	47,01	0,86	178,75	79,14	2987,48	2434,69	46,68	0,81	175,33	78,73	2983,64	2491,55	46,62	0,83	178,67	84,52	3107,48	2616,32	48,55
3048,61	2472,87	42,34	0,81	175,00	72,59	3131,50	2568,81	43,49	0,82	175,00	76,23	3181,40	2618,63	44,19	0,82	175,00	72,99	3085,42	2539,00	42,85
3147,78	2884,41	48,43	0,92	175,67	88,40	3208,21	3026,99	49,36	0,94	181,08	85,39	3000,76	2775,28	46,17	0,93	179,25	84,11	3040,35	2808,09	46,77
3130,26	2504,56	51,74	0,80	162,33	73,02	2993,56	2446,53	49,48	0,82	163,45	74,63	3077,50	2499,22	50,87	0,82	165,42	76,75	3175,85	2576,76	52,49
3062,34	2700,50	50,25	0,88	178,82	78,35	3015,27	2631,45	49,43	0,87	180,34	79,82	3004,28	2631,00	49,21	0,88	181,73	80,54	3029,66	2646,69	49,66
220,58	238,56	5,44	0,04	10,20	9,58	215,56	227,35	4,86	0,05	9,68	8,59	219,84	183,94	4,33	0,04	9,31	9,35	210,15	175,88	4,69

75%	75%	100%	100%	100%	100%	100%	100%		**MÉDIA**		MÉDIA
R	HR	VE	VO2	VCO2	VO2/Kg	R	HR	VO2max	VO2	%VO2max	HR
---	bpm	l/min	ml/min	ml/min	ml/min/Kg	---	bpm		ml/kg/min		bpm
0,9	149,0	55,4	2331,0	1998,7	35,3	0,9	150,1	54,93	34,31238	62,5%	147,0
0,9	186,1	64,7	2613,1	2435,2	40,8	0,9	185,3	52,00	40,42342	77,7%	182,5
0,9	176,8	74,1	2902,7	2518,9	53,8	0,9	177,8	61,42	53,85525	87,7%	174,5
0,9	197,4	68,0	2735,6	2393,9	48,9	0,9	197,4	68,06	49,03992	72,1%	194,7
0,9	165,1	61,6	2579,7	2323,8	46,1	0,9	169,6	60,69	48,3817	79,7%	167,0
0,9	168,1	66,0	2271,0	2064,6	40,6	0,9	168,3	50,22	40,78513	81,2%	165,5
0,8	153,6	59,1	2598,9	2102,9	40,6	0,8	152,5	52,57	40,30458	76,7%	153,0
0,9	160,0	46,8	2378,0	2023,6	33,0	0,9	159,8	44,45	31,99293	72,0%	150,2
0,8	177,1	78,8	3203,6	2650,9	49,3	0,8	179,2	52,52	47,50891	90,5%	174,9
0,8	170,6	70,4	2674,2	1983,9	44,6	0,7	165,9	52,20	46,30187	88,7%	162,2
0,87	170,4	64,5	2628,8	2249,7	43,3	0,86	170,6	54,9	43,3	78,9%	167,1
0,0	14,7	9,3	279,3	243,7	6,5	0,1	14,7	6,7	6,9	8,7%	15,0

75%	75%	100%	100%	100%	100%	100%	100%		**MÉDIA**		
R	HR	VE	VO2	VCO2	VO2/Kg	R	HR	VO2max	VO2		
---	bpm	l/min	ml/min	ml/min	ml/min/Kg	---	bpm		ml/kg/min		
0,89	176,67	71,12	3086,82	2693,14	46,77	0,87	179,58	54,93	45,1339	82,2%	173,5
0,85	174,08	61,60	2925,87	2462,07	45,72	0,84	177,25	52,00	45,51592	87,5%	172,6
0,94	183,92	79,94	2757,26	2490,42	51,06	0,90	184,17	61,42	52,49911	85,5%	182,6
0,92	197,08	68,78	2795,58	2469,81	49,92	0,88	194,50	68,06	52,74383	77,5%	192,6
0,91	179,75	69,20	2895,19	2655,08	51,70	0,92	184,00	60,69	52,1326	85,9%	178,6
0,81	167,83	58,40	2330,69	1968,72	41,62	0,85	171,67	50,22	41,73944	83,1%	167,9
0,83	157,67	65,58	2701,22	2187,86	42,21	0,81	158,75	52,57	42,53606	80,9%	159,0
0,84	170,58	57,10	2650,55	2241,88	36,81	0,85	168,67	44,45	38,39474	86,4%	166,5
0,94	177,67	83,74	3005,09	2805,42	46,23	0,93	179,33	52,52	46,14607	87,9%	177,6
0,83	165,00	87,70	2881,13	2474,72	48,02	0,86	165,00	52,20	47,2735	90,6%	165,0
0,88	175,03	70,32	2802,94	2444,91	46,01	0,87	176,3	54,9	46,41	84,7%	173,6
0,05	10,91	10,51	213,41	252,47	4,67	0,04	10,5	6,7	4,88	3,8%	9,7

75%	75%	100%	100%	100%	100%	100%	100%		**MÉDIA**		
R	HR	VE	VO2	VCO2	VO2/Kg	R	HR	VO2max	VO2		
---	bpm	l/min	ml/min	ml/min	ml/min/Kg	---	bpm		ml/kg/min		
0,89	185,67	88,20	3364,26	2997,77	50,97	0,89	188,75	54,93	50,43472	91,8%	183,0
0,91	187,42	66,58	2873,55	2603,78	44,90	0,91	186,42	52,00	45,76404	88,0%	182,1
0,86	184,70	97,72	3019,46	2588,33	55,92	0,86	183,55	61,42	57,46418	93,6%	178,2
0,88	200,00	95,59	3145,77	2845,19	56,17	0,90	203,17	68,06	55,98362	82,3%	200,2
0,92	184,33	78,63	2881,35	2703,83	51,45	0,94	186,50	60,69	52,9239	87,2%	180,7
0,90	183,75	84,03	2579,67	2307,31	46,07	0,89	185,33	50,22	45,43349	90,5%	178,5
0,84	181,17	84,72	3034,16	2530,18	47,41	0,83	180,92	52,57	47,20184	89,8%	176,8
0,82	175,00	76,50	3103,46	2519,60	43,10	0,81	175,00	44,45	43,14054	97,0%	175,0
0,92	171,33	81,25	3001,93	2786,15	46,18	0,93	163,00	52,52	47,71665	90,9%	174,8
0,81	167,55	76,00	3050,02	2555,47	50,41	0,84	169,33	52,20	51,03662	97,8%	163,6
0,88	182,09	82,92	3005,36	2643,76	49,26	0,88	182,20	54,9	49,71	90,9%	179,3
0,04	9,16	9,37	204,16	195,44	4,49	0,04	11,15	6,7	4,71	4,6%	9,2
