# Supplementary material for: Oxygen uptake kinetics and energy system’s contribution around maximal lactate steady state swimming intensity
Source: PLoS One. 2017 Feb 28;12(2):e0167263. doi: 10.1371/journal.pone.0167263 (PMC5330462; doi:10.1371/journal.pone.0167263)
Supplement: S2 File — (PDF) [file pone.0167263.s002.pdf]

| | 10min | | | | | | | | | TESTE ÚLTIMOS 20MIN - PÓS | | | | | | | | |
|---|---|---|---|---|---|---|---|---|---|---|---|---|---|---|---|---|---|---|
| | BASAL | A CARDIO | A1 | TD1 | TAU1 | A2 | TD2 | TAU2 | MRT | A BASAL | A CARDIO | A1 | TD1 | TAU1 | A2 | TD2 | TAU2 | MRT |
| **97,5% MLSS** | | | | (s) | (s) | (ml.kg.min-1) | (s) | (s) | s | (ml.kg.min-1) | (ml.kg.min-1) | (ml.kg.min-1) | (s) | (s) | (ml.kg.min-1) | (s) | (s) | s |
| **Swimmer 1** | 6,29 | 13,76 | 26,61 | 26,85 | 8,65 | 1,71 | 113,19 | 25,74 | 35,50 | 11,48 | 8,25 | 20,14 | 20,00 | 3,00 | 2,87 | 179,65 | 195,05 | 23,00 |
| **Swimmer 2** | 5,76 | 23,19 | 34,32 | 12,31 | 21,62 | 2,26 | 112,17 | 37,02 | 33,93 | 20,51 | 15,55 | 18,88 | 9,99 | 6,02 | 0,71 | 197,96 | 298,36 | 16,01 |
| **Swimmer 3** | 7,36 | 16,55 | 46,05 | 19,74 | 14,99 | 1,07 | 95,00 | 1,01 | 34,73 | 20,51 | 7,76 | 32,95 | 20,00 | 7,52 | | | | 27,52 |
| **Swimmer 4** | 7,04 | 15,37 | 43,18 | 9,94 | 14,86 | | | | 24,80 | 13,50 | 12,11 | 34,64 | 9,15 | 13,70 | 0,85 | 90,00 | 11,53 | 22,85 |
| **Swimmer 5** | 5,58 | 12,87 | 41,70 | 14,88 | 16,14 | 4,15 | 94,99 | 298,09 | 31,02 | 13,98 | 18,17 | 32,89 | 14,09 | 8,26 | 0,87 | 135,01 | 12,61 | 22,35 |
| **Swimmer 6** | 5,86 | 13,66 | 35,31 | 14,11 | 17,05 | | | | 31,16 | 23,73 | 5,43 | 15,98 | 9,99 | 13,77 | 0,84 | 100,74 | 257,99 | 23,76 |
| **Swimmer 7** | 12,74 | 6,90 | 28,01 | 7,84 | 26,31 | | | | 34,15 | 17,79 | 3,01 | 21,01 | 5,00 | 13,49 | 1,21 | 112,02 | 102,47 | 18,49 |
| **Swimmer 8** | 7,18 | 26,51 | 24,72 | 12,44 | 10,67 | | | | 23,11 | 7,11 | 15,04 | 23,67 | 6,63 | 8,57 | 1,73 | 195,02 | 298,05 | 15,20 |
| **Swimmer 9** | 7,74 | 12,69 | 37,28 | 14,34 | 20,80 | 2,49 | 105,00 | 58,27 | 35,14 | 19,85 | 11,23 | 26,99 | 15,85 | 17,56 | 1,28 | 200,00 | 299,99 | 33,41 |
| **Swimmer 10** | 6,27 | 22,28 | 40,04 | 10,00 | 12,17 | 1,41 | 97,26 | 0,15 | 22,17 | 11,03 | 7,24 | 36,21 | 9,12 | 15,89 | | | | 25,01 |
| **Média** | 7,2 | 16,4 | 35,7 | 14,2 | 16,3 | 2,2 | 102,9 | 70,0 | 30,6 | 15,9 | 10,4 | 26,3 | 12,0 | 10,8 | 1,3 | 151,3 | 184,5 | 22,8 |
| **DP** | 2,1 | 5,9 | 7,3 | 5,5 | 5,4 | 1,1 | 8,4 | 113,9 | 5,2 | 5,3 | 4,9 | 7,4 | 5,3 | 4,7 | 0,7 | 46,9 | 125,9 | 5,4 |

| | 10min | | | | | | | | | | | | | | | | | |
|---|---|---|---|---|---|---|---|---|---|---|---|---|---|---|---|---|---|---|
| | BASAL | A CARDIO | A1 | TD1 | TAU1 | A2 | TD2 | TAU2 | MRT | A BASAL | A CARDIO | A1 | TD1 | TAU1 | A2 | TD2 | TAU2 | MRT |
| **100% MLSS** | | | | (s) | (s) | (ml.kg.min-1) | (s) | (s) | s | (ml.kg.min-1) | (ml.kg.min-1) | (ml.kg.min-1) | (s) | (s) | (ml.kg.min-1) | (s) | (s) | s |
| **Swimmer 1** | 5,61 | 21,02 | 36,79 | 9,99 | 9,01 | 2,25 | 85,07 | 16,27 | 19,00 | 16,14 | 18,02 | 26,35 | 5,00 | 11,72 | 3,83 | 82,49 | 96,36 | 16,72 |
| **Swimmer 2** | 5,90 | 7,90 | 35,47 | 20,00 | 9,91 | 4,37 | 93,04 | 35,87 | 29,91 | 20,27 | 14,00 | 25,41 | 6,32 | 10,78 | | | | 17,10 |
| **Swimmer 3** | 8,24 | 13,71 | 43,62 | 15,03 | 14,40 | 2,60 | 155,02 | 25,55 | 29,43 | 25,40 | 5,17 | 26,21 | 9,19 | 11,43 | | | | 20,62 |
| **Swimmer 4** | 5,21 | 20,08 | 48,80 | 28,35 | 7,88 | | | | 36,23 | 19,94 | 8,59 | 31,87 | 9,80 | 9,77 | | | | 19,57 |
| **Swimmer 5** | 5,34 | 7,88 | 45,63 | 15,00 | 14,95 | 2,79 | 80,00 | 200,00 | 29,95 | 11,12 | 6,71 | 39,48 | 9,99 | 8,64 | 0,82 | 165,59 | 199,60 | 18,63 |
| **Swimmer 6** | 6,53 | 19,63 | 35,74 | 5,00 | 10,69 | | | | 15,69 | 16,72 | 15,05 | 24,39 | 14,99 | 3,87 | 0,84 | 193,25 | 299,85 | 18,86 |
| **Swimmer 7** | 6,17 | 8,56 | 36,08 | 15,61 | 18,77 | | | | 34,38 | 13,56 | 18,85 | 27,44 | 20,00 | 7,37 | 0,88 | 199,25 | 34,67 | 27,37 |
| **Swimmer 8** | 5,23 | 11,11 | 32,67 | 4,37 | 16,64 | 2,77 | 145,00 | 47,14 | 21,01 | 7,54 | 16,75 | 29,18 | 16,32 | 5,93 | 1,06 | 200,00 | 300,00 | 22,25 |
| **Swimmer 9** | 6,36 | 22,77 | 40,13 | 8,56 | 13,87 | 2,60 | 133,32 | 28,90 | 22,43 | 24,18 | 20,00 | 20,41 | 17,10 | 7,20 | 1,49 | 200,00 | 300,00 | 24,30 |
| **Swimmer 10** | 4,88 | 27,88 | 54,56 | 1,92 | 22,03 | | | | 23,95 | 21,52 | 18,58 | 31,73 | 10,48 | 20,23 | 1,45 | 80,00 | 0,89 | 30,71 |
| **Média** | 5,95 | 16,05 | 40,95 | 12,38 | 13,82 | 2,90 | 115,24 | 58,96 | 26,20 | 17,64 | 14,17 | 28,25 | 11,92 | 9,69 | 1,48 | 160,08 | 175,91 | 21,61 |
| **DP** | 0,97 | 7,13 | 7,02 | 8,07 | 4,53 | 0,75 | 32,98 | 69,87 | 6,78 | 5,72 | 5,43 | 5,22 | 4,92 | 4,47 | 1,07 | 55,20 | 131,42 | 4,59 |

| | 10min | | | | | | | | | | | | | | | | | |
|---|---|---|---|---|---|---|---|---|---|---|---|---|---|---|---|---|---|---|
| | BASAL | A CARDIO | A1 | TD1 | TAU1 | A2 | TD2 | TAU2 | MRT | A | A CARDIO | A1 | TD1 | TAU1 | A2 | TD2 | TAU2 | MRT |
| **102,5% MLSS** | | | | (s) | (s) | (ml.kg.min-1) | (s) | (s) | s | (ml.kg.min-1) | (ml.kg.min-1) | (ml.kg.min-1) | (s) | (s) | (ml.kg.min-1) | (s) | (s) | s |
| **Swimmer 1** | 6,81 | 10,95 | 39,70 | 10,00 | 16,96 | 4,49 | 80,05 | 72,66 | 26,96 | 21,87 | 17,76 | 27,51 | 4,95 | 8,74 | 1,61 | 185,01 | 78,11 | 13,69 |
| **Swimmer 2** | 6,36 | 10,38 | 36,52 | 14,93 | 13,80 | 3,74 | 110,00 | 36,68 | 28,73 | 23,67 | 6,20 | 21,36 | 6,84 | 20,38 | | | | 27,22 |
| **Swimmer 3** | 6,72 | 8,89 | 46,26 | 14,82 | 9,46 | 4,37 | 105,00 | 9,67 | 24,28 | 19,11 | 11,01 | 36,16 | 12,83 | 7,59 | | | | 20,42 |
| **Swimmer 4** | 6,97 | 25,32 | 51,36 | 5,91 | 18,61 | | | | 24,52 | 17,85 | 16,73 | 36,78 | 7,10 | 13,03 | | | | 20,13 |
| **Swimmer 5** | 5,19 | 8,50 | 47,12 | 5,00 | 16,68 | 2,91 | 87,57 | 128,61 | 21,68 | 27,85 | 17,79 | 23,40 | 10,02 | 1,42 | | | | 11,44 |
| **Swimmer 6** | 6,74 | 22,27 | 37,05 | 19,20 | 20,18 | 1,90 | 150,00 | 7,39 | 39,38 | 14,98 | 16,41 | 29,58 | 14,82 | 8,98 | 2,17 | 190,00 | 299,00 | 23,80 |
| **Swimmer 7** | 6,52 | 17,87 | 35,25 | 24,63 | 18,18 | 7,20 | 120,00 | 57,94 | 42,81 | 11,71 | 10,49 | 34,62 | 14,99 | 11,72 | 1,08 | 87,99 | 69,46 | 26,71 |
| **Swimmer 8** | 5,15 | 9,49 | 37,75 | 4,99 | 23,33 | 4,46 | 82,48 | 245,09 | 28,32 | 8,61 | 25,82 | 34,03 | 19,99 | 4,23 | | | | 24,22 |
| **Swimmer 9** | 7,76 | 13,64 | 39,24 | 5,00 | 19,16 | 6,10 | 92,86 | 96,34 | 24,16 | 22,07 | 9,34 | 24,57 | 7,17 | 12,74 | 0,76 | 199,99 | 24,18 | 19,91 |
| **Swimmer 10** | 5,98 | 23,25 | 42,37 | 9,99 | 3,32 | 5,14 | 80,23 | 144,22 | 13,31 | 20,50 | 17,40 | 30,18 | 11,84 | 7,50 | 1,82 | 172,80 | 64,80 | 19,34 |
| **Média** | 6,42 | 15,06 | 41,26 | 11,45 | 15,97 | 4,48 | 100,91 | 88,73 | 27,42 | 18,82 | 14,90 | 29,82 | 11,06 | 9,63 | 1,49 | 167,16 | 107,11 | 20,69 |
| **DP** | 0,80 | 6,54 | 5,35 | 6,81 | 5,80 | 1,59 | 23,23 | 75,79 | 8,46 | 5,76 | 5,67 | 5,53 | 4,72 | 5,25 | 0,57 | 45,33 | 109,26 | 5,15 |
